# Exposure to Violence Accelerates Epigenetic Aging in Children

**DOI:** 10.1038/s41598-017-09235-9

**Published:** 2017-08-21

**Authors:** Tanja Jovanovic, L. Alexander Vance, Dorthie Cross, Anna K. Knight, Varun Kilaru, Vasiliki Michopoulos, Torsten Klengel, Alicia K. Smith

**Affiliations:** 1 Department of Psychiatry and Behavioral Sciences, Emory University School of Medicine, 101 Woodruff Circle NE (Clifton RD NE), Atlanta, GA 30322 USA; 2 Genetics and Molecular Biology Program, 101 Woodruff Circle NE (Clifton RD NE), Atlanta, GA 30322 USA; 3 Neurobiology of Fear Laboratory, McLean Hospital, 115 Mill Street, Belmont, MA 02478 USA

## Abstract

Epigenetic processes, including DNA methylation, change reliably with age across the lifespan, such that DNA methylation can be used as an “epigenetic clock”. This epigenetic clock can be used to predict age and age acceleration, which occurs when methylation-based prediction of age exceeds chronological age and has been associated with increased mortality. In the current study we examined epigenetic age acceleration using saliva samples collected from children between ages 6–13 (N = 101). Children’s exposure to neighborhood violence and heart rate during a stressful task were assessed. Age acceleration was associated with children’s direct experience of violence (p = 0.004) and with decreased heart rate (p = 0.002). Children who were predicted to be older than their chronological age had twice as much violence exposure as other children and their heart rate was similar to that of adults. The results remained significant after controlling for demographic variables, such as sex, income and education. This is the first study to show the effects of direct violence exposure on epigenetic aging in children using salivary DNA. Although longitudinal studies are needed to determine whether accelerated epigenetic aging leads to adverse health outcomes later in life, these data point to DNA methylation during childhood as a putative biological mechanism.

## Introduction

Epigenetic processes, including DNA methylation, change reliably with age across the lifespan, such that DNA methylation can be used as an “epigenetic clock”^1, 2^. This epigenetic clock can be used to predict age and age acceleration, which occurs when the methylation-based prediction of age exceeds chronological age and has been associated with increased mortality^3^. While accelerated aging appears to be highly heritable^3^, there are significant environmental effects that can impact the epigenetic clock. Lifetime exposure to stress has been observed as a potential environmental mechanism for accelerated aging^4^. Studies in adults have found that cumulative lifetime stress^2^ and deployment trauma^5^ accelerate aging.

Childhood stress during development, such as institutional rearing^6^ and harsh parenting^7^, has also been associated with changes in methylation. Notably, the childhood stress samples from which DNA methylation was interrogated in the above studies were collected many years after the onset of the stressor. In the study that investigated the effects of institutional care in Romanian orphanages, the blood sample for epigenetic testing was collected at age 12^6^; in the study of harsh parenting in rural low socioeconomic status (SES) African-American families the sample was collected at age 20. In both cases the stress exposure was prolonged: decreased methylation was associated with the proportion of time spent in institutional care^6^ and accelerated aging was associated with changes in harsh parenting assessed at two time points prior to sampling (ages 11 and 16)^7^. In traumatized adult samples, early life stress does not appear to accelerate aging. In a study of Dutch veterans, deployment trauma but not childhood trauma predicted epigenetic aging^5^. A study of African-American individuals from a low SES, inner-city environment also found that cumulative life stress, but not childhood trauma, was associated with epigenetic aging^2^. These data suggest that accelerated epigenetic aging due to early life stress may be cumulative over an individuals’ lifetime and predictive of age-related disease^4^.

The proposed mechanism by which stress could impact epigenetic aging involves glucocorticoid receptors (GR), which are a critical component of the hypothalamic-pituitary-adrenal axis stress hormone system^4^. This hypothesis is supported by studies that have found gene-specific methylation changes in genes coding for GR function^8, 9^ and by changes induced by glucocorticoid administration^2^. While some studies have focused on methylation changes in CpG sites of specific stress-related genes^6^, others have found that global epigenetic changes in genome-wide methylation are also associated with life stress exposure^2^. The extent to which accelerated aging is tissue specific is currently unknown, and most of the studies to date have examined blood cells in adults^4^. It is of critical importance to test whether the same patterns are observed in more noninvasively collected samples, such as saliva. Furthermore, only one study to date has examined the effects of stress on DNA methylation in children, and whether the stress was still ongoing at the time of sampling is unclear^6^. Finally, the exact nature of the stressor has varied across studies. While most studies have examined stressful life experiences, very few have examined traumatic experiences^4^, which appear to have a rapid effect on accelerated aging^5^. One study compared direct personal stress exposure to the stress of one’s social network and found that epigenetic aging was predicted only by personal stress, indicating that direct exposure may be critical^2^.

The physiological mechanism by which accelerated aging might increase mortality is unclear to date. While stress and trauma are known to induce illness, such as metabolic syndrome and cardiovascular disease^10, 11^, and accelerated aging is associated with increased mortality from cardiovascular disease^12^, the mechanisms for these associations are not well understood. One study of combat veterans found that post-traumatic stress disorder (PTSD) symptoms were associated with epigenetic aging, which in turn was associated with decreased neural integrity predictive of aging-related cognitive decline^13^. However, other physiological outcomes such as heart rate and heart-rate variability under stress, have not been examined with respect to trauma and accelerated aging. The current study aimed to address some of these gaps in the literature, by assessing epigenetic aging in children ages 6–13 using saliva samples.

We hypothesized (1) that higher levels of experienced trauma would result in accelerated epigenetic aging and (2) that cardiovascular changes would be predicted by epigenetic age. The children were recruited from a highly traumatized urban population and reported on their directly experienced as well as witnessed exposure to neighborhood violence. Additionally, we examined heart rate and heart-rate variability under a stressful condition to examine possible effects on cardiovascular markers. We found that direct violence exposure was associated with accelerated epigenetic aging and decreased heart rate.

## Results

**Figure 1:**
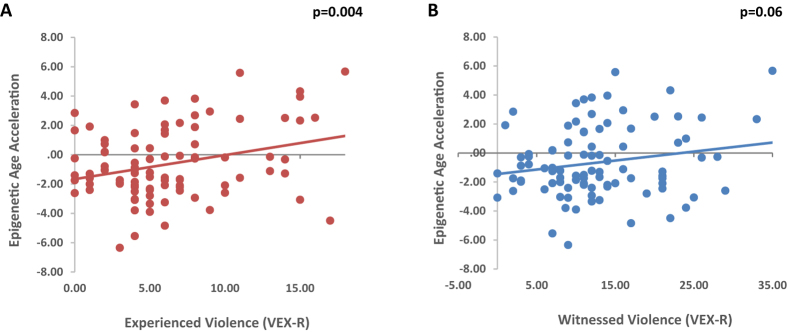
Scatter plots and regression lines for the association (**A**) between age acceleration and experienced violence (r = 0.30, p = 0.004), and (**B**) between age acceleration and witnessed violence (r = 0.19, p = 0.06). Age acceleration was defined by taking the residual of the linear regression of DNAm age on chronological age.

The sample demographics and levels of violence exposure are shown in Table 1. Most of the sample was low income (90.9% with less than $2,000/month) and over half of the mothers had a high school degree or less (Table 1). Each child experienced an average of six traumatic events and witnessed on average twice as many as that, suggesting a high level of violence exposure. However, the children had presented with low to moderate levels of internalizing or externalizing symptoms reported by their mothers on the BASC-2. In an exploratory epigenome-wide association study analysis, 200 CpG sites associated with violence exposure (9.1E-7 < p < .05), but none remained significant after correction for multiple comparisons (Supplemental Figure S1). DNAm age was robustly correlated with chronological age (r = 0.46, p = 0.000001, see Supplemental Figure S2). Age acceleration (the residual between DNAm age and chronological age) was positively correlated with total score on the VEX-R, r(93) = 0.25, p = 0.02. When we separately examined the frequency scores for directly experienced violence and witnessed violence, the association was even stronger for direct experiences (r = 0.30, p = 0.004), but was not significant for witnessed violence (r = 0.19, p = 0.06). The correlation between experienced violence and accelerated aging remained significant after adjusting for epithelial cell proportion in the sample (R^2^ = 0.107, F_change_ = 4.49, p < 0.05). Figure 1 shows a scatter plot for the association between accelerated aging and each measure of violence exposure. A linear stepwise regression controlling for demographic factors, with child sex, parental education, and household income entered into the first step, showed that experiencing violence predicted 8.5% of the variance in age acceleration beyond the other variables (R^2^ = 0.085, F_change_ = 9.15, p = 0.003). The demographic data entered in the first step were also predictive of accelerated aging (R^2^ = 0.12, F_change_ = 3.95, p = 0.01), suggesting that low SES predicted epigenetic aging; however, the violence exposure independently accelerated aging. Notably, internalizing and externalizing scores on the BASC-2 were not correlated with accelerated age or DNAm age, suggesting that the association between age acceleration and trauma is independent of current behavioral symptoms. There were also no sex differences in DNAm age or accelerated aging.

**Table 1:** Demographic and clinical data for the sample.

**AGE (n = 101)**	
mean in years (range)	9.73 (6–13)
**SEX (n = 101)**	
% female	54.5%
**INCOME (n = 99)**	
% under $1000/mo	56.6%
% between $1000–2000/mo	34.3%
% over $2000/mo	9.1%
**EDUCATION (n = 99)**	
% high school or less	57.7%
% some college/technical	27.3%
% college/grad school	5.0%
**VIOLENCE EXPOSURE (n = 93)**	
VEX-R TOTAL (mean, SD)	18.92 (10.9)
VEX-R Experienced (mean, SD)	6.13 (4.4)
VEX-R Witnessed (mean, SD)	12.79 (7.5)
**SYMPTOMS (n = 95)**	
BASC-2 Internalizing (t-score)	53.46
BASC-2 Externalizing (t-score)	57.02

The startle paradigm was mildly stressful, as heart rate increased on average by two beats per minute (BPM) and heart-rate variability decreased from the light to the dark phase of the experiment (F = 4.25, p = 0.04 and F = 5.40, p = 0.02, respectively). Linear regressions controlling for demographic data and epithelial cell proportion found that accelerated aging (R^2^ = 0.09, F_change_ = 6.89, p = 0.01) and DNAm age (R^2^ = 0.15, F_change_ = 12.61, p = 0.001) predicted lower HR in the dark phase. HRV was not associated with accelerated aging or DNAm age. Neither experienced nor witnessed violence exposure were significantly associated with HR or HRV during the dark-enhanced startle task. When violence exposure was added to the linear regression, DNAm age remained predictive of HR (R^2^ = 0.08, F_change_ = 6.33, p = 0.01), even after controlling for chronological age (R^2^ = 0.06, F_change_ = 4.59, p = 0.04), which itself was not associated with HR.

**Figure 2:**
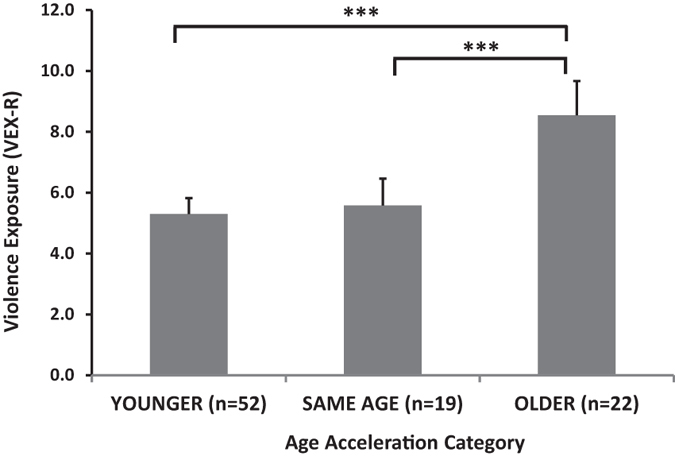
Frequency of directly experienced violence (VEX-R) across the accelerated age groups. The epigenetically oldest group, +1 residual of the age acceleration, had almost twice as much violence exposure than the other groups. An ANOVA showed a significant effect of group on levels of experienced violence, F(2,92) = 10.59, p < 0.001, post-hoc tests ***p < 0.01. Abbreviations: VEX-R, Violence Exposure Scale for Children Revised.

In addition, we categorized children according to age acceleration into three groups based on the residual values: a “younger” group that had negative age acceleration (up to −1 residual), a “same age” group that ranged between −1 and 1 residual, and an “older” group with positive age acceleration residual of 1 and above. An ANOVA showed a significant effect of group on levels of experienced violence, F(2,92) = 10.59, p < 0.001, with post-hoc tests showing that the positive age acceleration (i.e., “older”) group had significantly more trauma than the other two groups (both p < 0.001), Fig. 2.

**Figure 3:**
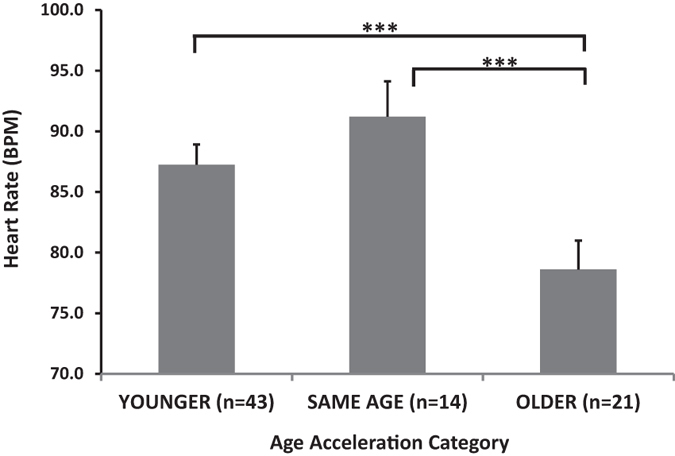
Heart rate during the dark phase of the dark-enhanced startle test across age acceleration groups. ANOVA showed a main effect of age acceleration category on HR, F(2,77) = 6.59, p = 0.002. Post-hoc tests showed that the older groups had significantly lower HR than the other two groups. ***p < 0.01. Abbreviations: HR, Heart rate.

We then examined HR across the acceleration age categories, defined by residuals as above. There was a main effect of age acceleration category on HR, F(2,77) = 6.59, p = 0.002. Post-hoc tests showed that the positive age acceleration, or “older” group had lower HR than the other two groups (Fig. 3).The average HR for this group (76.2 BPM) was 10 beats per minute lower than the “younger” groups (87.2 and 91.2, post-hoc group comparison p = 0.004 and p = 0.001, respectively), and was much closer to the average HR (72.9 BPM) for adults (ages 18–30) sampled from the same population using the same experimental paradigm (data taken from dataset published by Kamkwalala and colleagues)^14^. Taken together these results suggest that age acceleration is associated with decreased HR, which is more adult-like in the dark-enhanced startle paradigm. Notably, other startle responses, such as potentiation of the eyeblink electromyography, did not differ between age acceleration groups during the dark-enhanced startle task F(2,78) = 0.62, p = 0.54.

## Discussion

To our knowledge this is the first study to examine the effects of violence exposure on salivary measures of epigenetic aging in children. Based on previous studies of trauma and accelerated epigenetic aging in adults^5^, we hypothesized that higher frequency of traumatic experiences would be associated with increased epigenetic aging. We found this to be true in that experienced, but not witnessed, violence was associated with greater DNAm age acceleration. In fact, children who were predicted to be in an older epigenetic age group had experienced almost twice as much violence as the other children. This finding is consistent with the literature in adults^2, 5^, in that higher cumulative stress and trauma increased epigenetic aging. Studies of early institutional care^6^ and changes in harsh discipline^7^ have found similar results in youth and young adults using DNA methylation from blood samples. The children in our sample were recruited from a low SES population; because low income has been shown to accelerate epigenetic aging^15^, we controlled for household income and parental education in the analyses and found that violence exposure was predictive of accelerated epigenetic aging beyond the low SES variables.

We additionally examined whether epigenetic aging would be associated with autonomic function during a stressful task, in order to see whether accelerated epigenetic aging may lead to cardiovascular risk. We hypothesized that epigenetic age would be associated with heart rate and heart-rate variability during a dark-enhanced startle task. We found that accelerated epigenetic aging and DNAm age were negatively correlated with HR, but not HRV, in the dark phase. This suggests that those with higher DNAm age acceleration showed decreased HR to the stressful task. While we would expect that increased HR might be a risk factor for cardiovascular disease, it is important to note that these children were young and medically healthy. The decrease in HR may not have been maladaptive, however it is consistent with age acceleration. The children who demonstrated epigenetic age acceleration, and were predicted by DNAm age to be older than their chronological age, had HR levels similar to adults and much lower than other children. The epigenetically older children did not show blunted eyeblink startle responses in the task, therefore the lower HR was unlikely due to them being less startled, or less anxious during the dark phase of the task.

It is possible that age acceleration is an adaptive response to traumatic or stressful environments in the short-term. An adult-like epigenetic and physiological “setting” of the cardiovascular system may be beneficial to overcome or deal with stressors and it may be a normal reaction to lower the HR in response to a mild stressor instead of an overshooting cardiovascular reaction. The decreased HR in the epigenetically older children may also be reflective of emotional adaptation, i.e. it is possible that the stressor did not evoke as strong a reaction on those children as in the epigenetically younger ones.

While there may be adaptive or even beneficial effects of accelerated aging with respect to trauma exposure, there may be long-term physiological costs associated with accelerated aging that can lead to cardiovascular disease later in life. Therefore, there may be a short-term “trade off” for accelerated aging that could only be observed in long-term longitudinal studies.

Trauma-related disorders, such as PTSD, have been associated with increased cardiovascular risk^11^ and increased HR^16, 17^. Women with a history of childhood abuse also show increased HR to a stressful task^18^. In our study, however, we did not find an association between trauma exposure and HR in children, and epigenetic aging appeared to be an independent predictor of HR. We also did not find that accelerated epigenetic aging was associated with early signs of pathology on the BASC-2, which is also consistent with some studies of PTSD symptoms^5^, but not others^13^. It is possible that the pathological effects (both for mental and physical health) are long-term outcomes of accelerated aging and that the children we studied were not yet manifesting those effects. Longitudinal studies are needed to determine the exact health outcomes of accelerated epigenetic aging and decreased HR in these children; due to the cross-sectional nature and limited age range in the current study, we were not able to observe such outcomes. Therefore, in the current study we are not able to conclude that the effects of violence exposure on aging lead to adverse outcomes. However, because the effects of stress and trauma on accelerated aging may be reversible if there are timely interventions^4^, identifying these early biomarkers of accelerated aging may be critical to preventing these diseases later in life. In fact, social interventions in human studies^7^ and molecular interventions in animal studies^19^ have shown that epigenetic changes can be reversed.

One of the limitations of the study is the cross-sectional sample of children so that all age-related and epigenetic changes were compared between rather than within individuals. Longitudinal data are needed in order to address the permanence of epigenetic aging effects. Furthermore, violence exposure information was collected via child self-report, which has several inherent biases, including recollection bias. It is possible that early trauma exposure the child does not remember could also impact epigenetic aging. Replication samples using more objective measures of violence and trauma exposure in children, such as Child Protective Services reports, would provide further support for these findings. This is the first study that we are aware of that has examined the impact of violence exposure on epigenetic aging in children; given the implications of accelerated aging on risk for negative health outcomes this relationship should be explored more in future studies. Finally, there may be specific gene variants that affect the epigenetic clock; for instance, genome-wide methylation analyses that found epigenetic aging in cerebellar tissue overlapped with genes associated with aging disorders such as Alzheimer’s and Parkinson’s^20^.

In summary, one of the main contributions of this work was to show that DNAm age assessed in saliva of children demonstrates accelerated aging with increased violence exposure. In addition, accelerated epigenetic age is associated with adult-like autonomic functioning in children with violence exposure.

## Methods

### Participants

Study participants were 101 African American children between 6 and 13 years of age (mean = 9.73, SD = 1.67 years), whose mothers were recruited from the waiting rooms of primary care clinics in an urban hospital in Atlanta, GA. The participants were recruited as part of an ongoing longitudinal study of trauma exposure in children. A subset of data from this study has been published previously^21, 22^; the current study, however, only included individuals who had DNA methylation data at the first study visit. In order to be eligible for the study, child participants had to be between 6 and 13 years of age and willing to participate; exclusion criteria were a diagnosis of autism spectrum disorders, bipolar or psychotic disorders, or cognitive impairment. Mothers were excluded if they had current psychosis or a positive urine test for substance use. Prior to their participation, all mothers gave written informed consent as well as parental permission for their children, and the children provided study assent approved by the Emory University Institutional Review Board and the Grady Hospital Research Oversight Committee. All procedures were conducted in accordance with IRB guidelines and regulations.

### Clinical Assessments

Violence exposure in children was assessed using the Violence Exposure Scale for Children-Revised (VEX-R)^23^. The VEX-R is a 22-item cartoon-based self-report interview of children’s lifetime exposure to violence. The VEX-R has 9 questions that capture frequency of direct violence exposure (for example “How many times has someone pointed a knife or real gun at you?”) and 13 questions that measure frequency of witnessing violent events (for example “How many times have you seen a person stab another person with a knife?”). The VEX-R generates a total frequency score as well as separate scores for experienced and witnessed violence.

To characterize the sample in terms of psychological symptoms and potential diagnoses, the Behavior Assessment System for Children – Second Edition (BASC-2)^24^ was administered to children. The BASC-2 yields several clinical and adaptive continuous subscale scores normalized by child age and gender in a non-clinical, normative sample. For the present study, we included the internalizing and externalizing composite score based on parent report because these areas are common trauma-related sequelae in children^25^. Test-retest reliability, internal consistency, and convergent validity of the scales are very high^24^.

### DNA Methylation

DNA was extracted from saliva in Oragene collection vials (DNA Genotek Inc, Ontario, Canada) using the DNAdvance kit (Beckman Coulter Genomics, Danvers, MA). Approximately 2 ml of saliva was collected from each participant. Our previous research comparing methylation in brain, blood and saliva found that saliva produced methylation data comparable to brain tissue^26^. Genomic DNA was bisulfite converted using the EZ-96 DNA Methylation Kit (Zymo Research), and DNA methylation levels were interrogated for >480,000 CpG sites using the Illumina HumanMethylation450 BeadChip. Hybridization and processing was performed according to the manufacturer’s instructions as previously described^27^. Beta values were generated with BeadStudio and were set to missing (no call) if detection p-values exceeded 0.001. All samples had probe detection call rates <95% and an average intensity value of either <50% of the experiment-wide sample mean or <2,000 arbitrary units (AU). CpG sites with missing data for >10% of samples were excluded from analysis. Normalization of probe distribution and background differences between Type I and type II probes was conducted using Beta Mixture Quantile Normalization (BMIQ)^28^, and ComBat was used to account for sources of technical variations including batch and positional effects^29^.

### Heart Rate

The physiological data were acquired using Biopac MP150 for Windows (Biopac Systems, Inc., Aero Camino, CA). The acquired data were filtered, rectified, and smoothed using MindWare software (MindWare Technologies, Ltd., Gahanna, OH) and exported for statistical analyses. Heart rate (HR) and heart-rate variability (HRV) data were acquired using the electrocardiogram (ECG) and respiration (RESP) modules of the Biopac system. The ECG signal was amplified by a gain of a 1000, filtered with a Hamming windowing function, and with a 60 Hz notch filter. ECG was recorded using two disposable Ag/AgCl electrodes pre-coated with electrolyte gel; one was placed on the right side of the upper torso, 1 cm below the clavicle, and the second on the inside of the left wrist. Respiration was measured using a measured with a chest band transducer. HRV was quantified during one minute intervals by spectral analysis of the time-sampled interbeat interval series, according to the methods recommended by the Society for Psychophysiological Research Committee on Heart Rate Variability^30^. High-frequency HRV was sampled from 0.12 to 0.40 Hz, and transformed by natural log.

The HR/HRV data were collected during a startle paradigm described in previous studies^31^. The dark-enhanced startle task consisted of two blocks each with eight startle probes. The startle probes were 40ms bursts of 104 dB white noise delivered through headphones. In each block, four startle probes were delivered in the dark phase and four were delivered in the light phase. The order of light and dark were counterbalanced across subjects. The lights in the startle booth were controlled by a timer which was synchronized with the presentation of the startle probes. Across the entire experimental session, inter-trial intervals ranged from 9 to 22 seconds.

### Data Analyses

DNA methylation-based age prediction (DNAm age) was performed using the R code and statistical pipeline developed by Horvath^1^. The age acceleration metric was developed using 50,000 tissues across the body and from different age samples in order to result in a universal and reliable measurement which would not differ in accuracy by tissue. The average epigenetic age (DNAm age) of our sample was 9.50 (SD, 2.59) and highly correlated with chronological age (9.72, SD = 1.67), p < 0.0001. Age acceleration was defined by taking the residual of a linear regression of DNAm age on chronological age. Though the DNAm age predictor developed by Horvath was trained on multiple tissues, the proportion of epithelial (buccal) cells and leukocytes in saliva varies between individuals and can be a confounding factor^26^. We used the method described by Houseman and colleagues, which uses DNA methylation from a reference tissue, to estimate the proportion of epithelial (buccal) cells in a heterogeneous population of cells such as saliva^32, 33^. We identified an appropriate reference from buccal cells (GSE46573) from the Gene Expression Omnibus (GEO). The proportion of epithelial cells in each sample was included as a covariate in all association tests. An exploratory epigenome-wide association study was also conducted. For each CpG site, methylation proportion was modeled as a linear function of violence exposure, adjusting for age, sex, cellular heterogeneity. To account for multiple comparisons, the false discovery rate (FDR) was controlled at 5%.

Violence exposure was correlated with age acceleration using the VEX-R total score, as well as the experienced and witnessed subscores of the VEX-R. Significant correlations were followed up by linear regressions controlling for demographic variables such as sex, household income and parental education. Categorical analyses of DNAm age groups were conducted using analysis of variance (ANOVA), with post-hoc tests comparing groups.

The HR and HRV data during the dark-enhanced task were analyzed in a three-way mixed ANOVA, with Block (two levels) and Phase (light, dark) as repeated measures factors with HR and HRV as dependent variables. HR data was also correlated with age acceleration and DNAm age and compared across age categories using ANOVAs. Methylation analyses were conducted in R and further statistical analyses were performed using SPSS 20.0 for Windows, and alpha was set to 0.05.

## Electronic supplementary material

Supplemental Information
