# Supplementary material for: Exposure to Violence Accelerates Epigenetic Aging in Children
Source: Sci Rep. 2017 Aug 21;7:8962. doi: 10.1038/s41598-017-09235-9 (PMC5566406; doi:10.1038/s41598-017-09235-9)

Supplemental Information

Exposure to Violence Accelerates Epigenetic Aging in Children

Tanja Jovanovic, PhD[1], L. Alexander Vance, MA[1], Dorthie Cross, PhD[1], Anna K Knight, BS[2], Varun Kilaru, PhD[1], Vasiliki Michopoulos, PhD[1], Torsten Klengel, PhD[3], Alicia K. Smith, PhD[1]

SREP-16-47171A

[1]Department of Psychiatry and Behavioral Sciences
Emory University School of Medicine
101 Woodruff Circle NE (Clifton RD NE)
Atlanta, GA 30322

[2]Genetics and Molecular Biology Program
101 Woodruff Circle NE (Clifton RD NE)
Atlanta, GA 30322

[3]Neurobiology of Fear Laboratory
McLean Hospital
115 Mill Street
Belmont, MA 02478

Corresponding Author:
Tanja Jovanovic, PhD
Department of Psychiatry & Behavioral Sciences
Emory University School of Medicine
49 Jesse Hill Jr Dr
Atlanta, GA 30303, USA
+1 404 778 1485
tjovano@emory.edu

Running title: Epigenetic Aging in Children

Figure S1. DNA methylation differences associated with violence exposure. On this Manhattan plot, the x-axis is the position of each CpG site by chromosome, and the y-axis is the negative $\log_{10}$ of the p-value for association of each CpG site with violence exposure. The black line indicates experiment-wide significance.

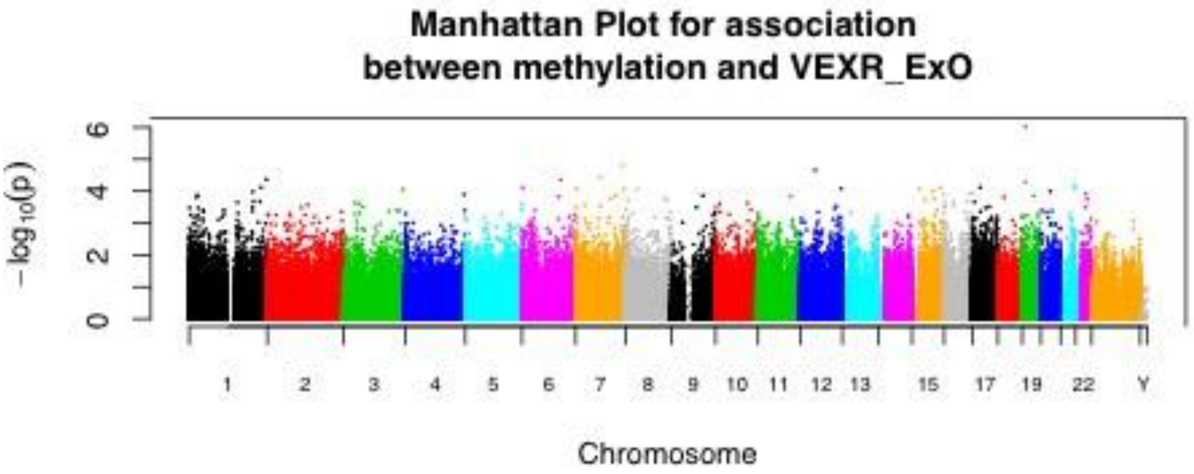

Figure S2. Scatter plot of DNAm Age and Chronological Age

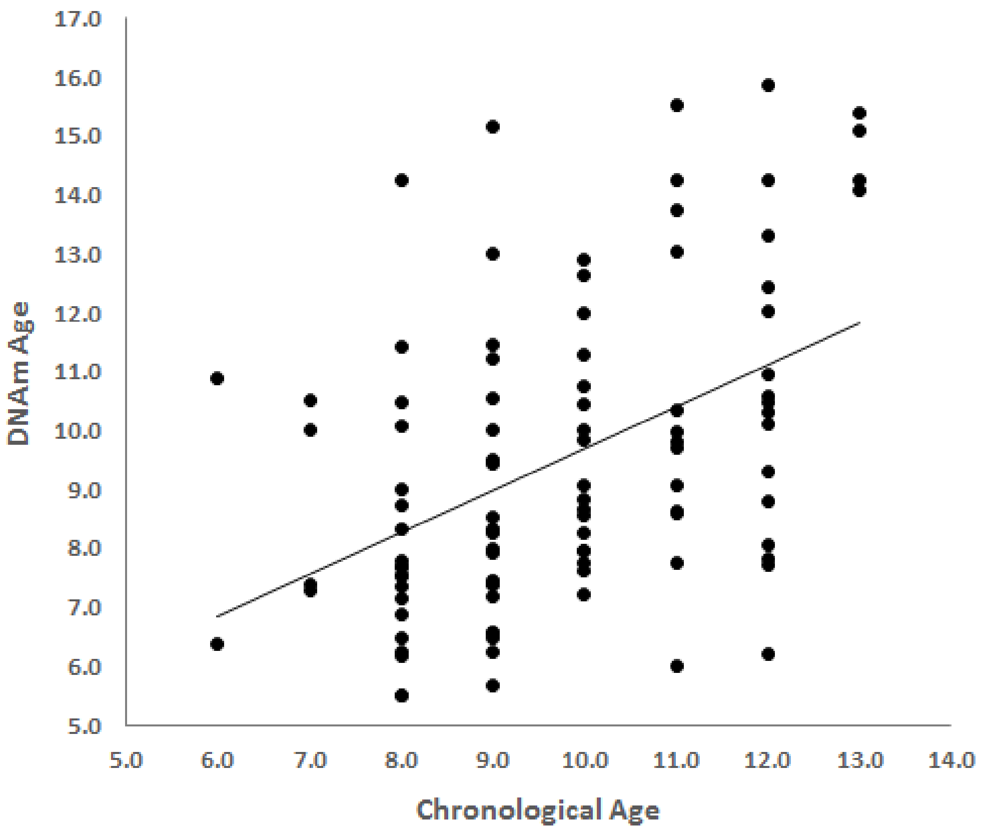

Supplement: Supplementary file 1 — Supplemental Information [file 41598_2017_9235_MOESM1_ESM.pdf]
